# Harmonizing Iron Terminology and Nomenclature

**DOI:** 10.7759/cureus.97362

**Published:** 2025-11-20

**Authors:** Abdulqadir J Nashwan, Badir Zakir, Yousef Hawas, Mohamed Yassin

**Affiliations:** 1 Nursing and Midwifery Research, Hamad Medical Corporation, Doha, QAT; 2 Surgery, NES Healthcare, Leicester, GBR; 3 Medical Education and Simulation, Tanta University, Tanta, EGY; 4 Department of Hematology, National Centre for Cancer Care and Research, Hamad Medical Corporation, Doha, QAT

**Keywords:** anaemia of inflammation, iron deficiency, iron metabolism, iron nomenclature, standardization

## Abstract

Iron is indispensable to human physiology, yet its terminology remains fragmented across clinical, laboratory, and chemical fields. With recent updates from several clinical guidelines, a unified framework for iron nomenclature has emerged. This editorial advocates for harmonization, replacing outdated and inconsistent terms such as functional iron deficiency and ferrous/ferric with standardized equivalents like iron-restricted erythropoiesis and iron(II)/iron(III). Consistent terminology is not a matter of semantics but of precision, ensuring clarity in communication, comparability across studies, and accuracy in diagnosis. Adoption of a common lexicon will strengthen research interpretation, education, and global clinical practice.

## Editorial

**Figure 1:**
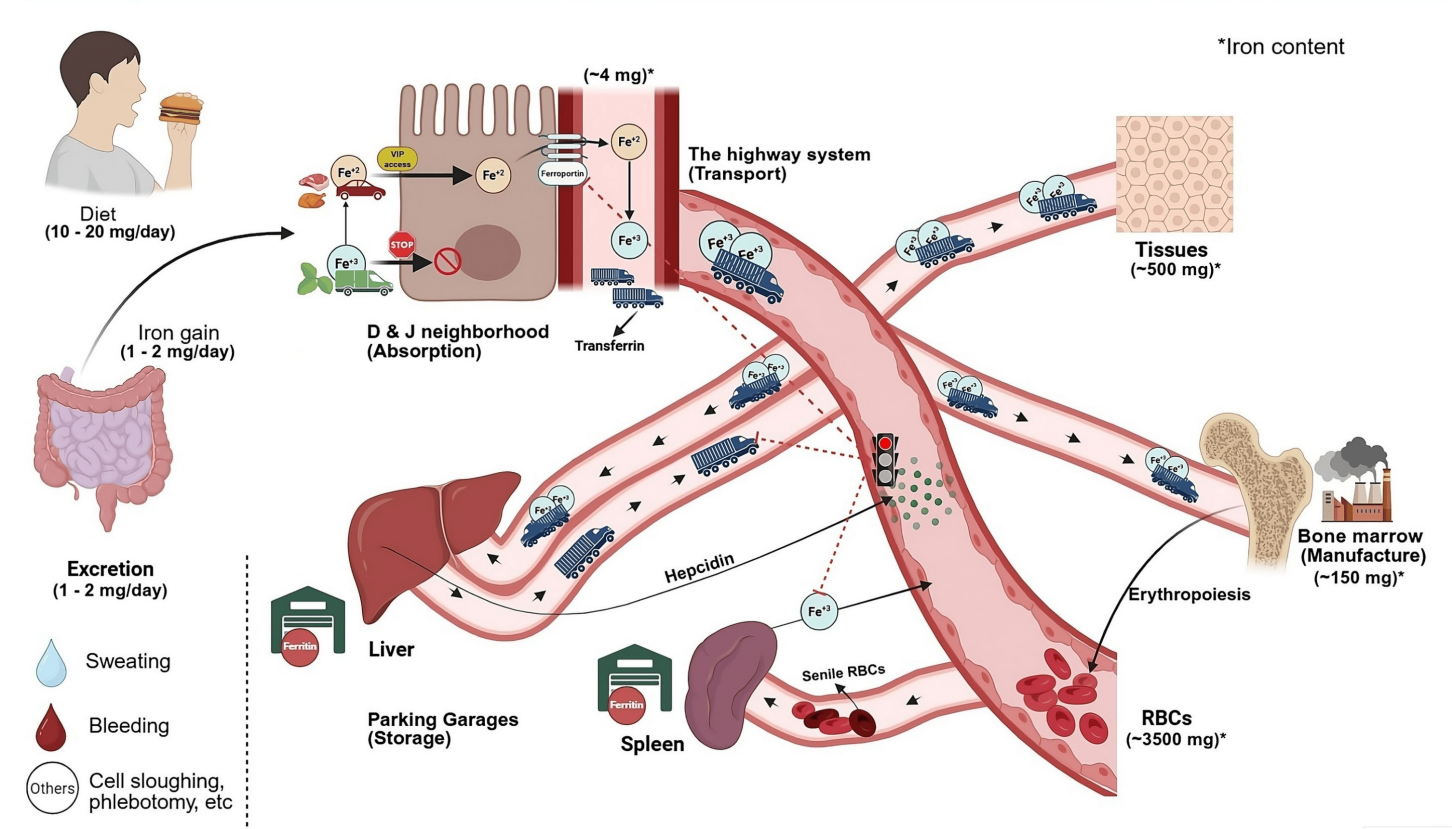
Mechanisms and distribution of iron absorption, transport, storage, and utilization in the human body This schematic illustrates the major physiological pathways and compartments involved in iron homeostasis. Dietary intake and absorption: Iron is ingested through the diet (10–20 mg/day), with a net gain of 1–2 mg/day due to regulated intestinal absorption. Iron uptake occurs primarily in the duodenum and jejunum (D&J neighborhood), where Fe²⁺ ions are absorbed by enterocytes via specialized transporters. Transport: Absorbed iron enters the bloodstream (~4 mg in circulation) and binds to transferrin for systemic transport (the highway system). Iron is delivered to various tissues (~500 mg stored in tissues), the bone marrow (~150 mg), and RBCs (~3500 mg). Storage: Excess iron is stored as ferritin in the liver and spleen (“parking garages”). Regulatory mechanisms: Hepcidin, produced by the liver, plays a pivotal role in iron balance by inhibiting ferroportin, regulating transferrin production, and Fe+3 produced by the spleen, thus controlling iron overload and overall systemic iron availability. Erythropoiesis and recycling: The bone marrow manufactures RBCs using iron. Senile RBCs are phagocytosed by the spleen, and iron is recycled back into circulation. Excretion and loss: Iron loss occurs through excretion (1–2 mg/day) via sweating, bleeding, cell sloughing, phlebotomy, and other routes. Iron content values for major compartments are indicated with asterisks (*). This figure has been created by the co-author, Yousef Hawas, using BioRender.

Iron is an essential micronutrient that underpins critical physiological processes, including oxygen transport, mitochondrial energy production, DNA synthesis, and immune function [1,2]. Most body iron is incorporated into hemoglobin and myoglobin to facilitate oxygen delivery. At the same time, smaller but vital pools of heme and iron-sulfur clusters exist in heme and iron-sulfur cluster proteins that drive enzymatic reactions and cellular respiration [3]. Because the human body lacks a regulated mechanism for iron excretion, systemic iron balance relies on coordinated absorption, recycling, and storage (Figure 1), tightly controlled by the hepatic hormone hepcidine, which regulates iron release through its receptor, ferroportin [2,3]. Dysregulation of this system can lead to iron deficiency, resulting in anemia and impaired tissue function, or iron overload, causing oxidative damage and organ injury [1,4].

Iron is fundamental to human physiology, yet the language used to describe its metabolism has varied widely across disciplines and eras. Clinical medicine has traditionally used terms such as absolute and functional iron deficiency, while older literature relied on ferrous and ferric chemical designations. Laboratory indices have also suffered from inconsistent use, with terms such as Transferrin Saturation Index (TSI) and Transferrin Saturation (TSAT) often used interchangeably. Such variability risks misinterpretation, limits cross-study comparability, and complicates the translation of research into practice.

Inconsistent terminology is not merely a linguistic issue-it carries profound clinical implications. Misinterpretation of terms such as “functional iron deficiency” versus “iron-restricted erythropoiesis” may lead to inappropriate therapeutic interventions, including unwarranted intravenous iron administration or missed diagnosis of anemia of inflammation. For instance, patients with chronic kidney disease or heart failure could be erroneously classified as iron-deficient despite adequate stores if older criteria are applied. This diagnostic ambiguity affects patient outcomes, healthcare costs, and the comparability of intervention trials. Hence, harmonizing terminology related to iron is a necessary step toward improving clinical decision-making and ensuring evidence-based practice.

With the publication of the Kidney Disease: Improving Global Outcomes (KDIGO) 2025 guidelines [5], The International Society for the Study of Iron in Biology and Medicine (BIOIRON) consensus statements (2022)[6], and the recommendations of the International Union of Pure and Applied Chemistry (IUPAC)[7], There is now an opportunity to harmonize this terminology. This editorial synthesizes these sources and presents an updated framework for iron nomenclature that bridges clinical, laboratory, and chemical perspectives.

The problem is not trivial, as the burden of iron-related disorders is immense. In clinical practice, iron deficiency anemia remains the leading cause of anemia worldwide, accounting for nearly half of cases [8]. However, the interpretation of iron studies varies significantly between laboratories and guidelines. Inconsistencies in the terminology hinder communication among healthcare providers [9].

Terminology drift also limits research synthesis; for instance, a study describing functional iron deficiency in 2005 may not align with another using iron-restricted erythropoiesis in 2025, even if both represent the same pathophysiological process. Without a shared framework, cross-study comparability suffers, and guidelines risk ambiguity.

The updated nomenclature clarifies and, in some cases, replaces outdated terms. For example, the former term "absolute iron deficiency" is now called "systemic iron deficiency," while "functional iron deficiency" is more accurately described as "iron-restricted erythropoiesis." Similarly, older chemical descriptors such as ferrous and ferric are replaced by the IUPAC-preferred iron(II) and iron(III) [7].

Recent international consensus has led to the establishment of a unified nomenclature for iron metabolism, bridging clinical, laboratory, and chemical disciplines [5-7,10]. The new framework introduces standardized terminology that replaces outdated and inconsistent expressions long used in clinical practice and research. For instance, the term “absolute iron deficiency” has been refined to “systemic iron deficiency”, denoting a state in which ferritin levels are below 100 ng/mL and/or transferrin saturation (TSAT) is less than 20%, reflecting inadequate systemic iron availability. Similarly, “functional iron deficiency” is now referred to as “iron-restricted erythropoiesis”, a condition characterized by normal or elevated ferritin but low TSAT due to hepcidin-mediated iron sequestration that limits erythropoiesis despite sufficient total body iron stores.

In cases of iron excess, “hemochromatosis” and “siderosis” have been consolidated under the term “iron overload”, which is classified as either primary (hereditary) or secondary (for example, transfusion-related). Diagnosis now relies on the integrated interpretation of ferritin, TSAT, and MRI T2* findings. The previously used “labile plasma iron” has been replaced by “non-transferrin-bound iron (NTBI)”, describing a circulating, redox-active iron fraction that contributes to oxidative stress and tissue injury in overload states.

From a chemical perspective, the IUPAC-recommended notations iron(II) and iron(III) have replaced the traditional ferrous and ferric labels, ensuring consistency with international chemical standards. Similarly, the laboratory measure transferrin saturation (TSAT) has been standardized as the ratio of serum iron to total iron-binding capacity (TIBC), expressed as a percentage, replacing the older term transferrin saturation index (TSI). Finally, “anemia of inflammation” is now preferred over “anemia of chronic disease”, emphasizing the role of inflammatory cytokines and hepcidin in restricting iron mobilization despite adequate stores. Table 1 summarizes these revised terms, along with their former usage and definitions for reference.

**Table 1:** Evolution of iron-related nomenclature

Former Name	New / Preferred Name	Definition	Key References
Iron deficiency	Iron Deficiency (ID)	Depleted systemic iron stores, regardless of anemia status.	[5,8]
Iron deficiency anemia	Iron Deficiency Anemia (IDA)	Iron deficiency with reduced hemoglobin concentration below the WHO thresholds.	[8]
Absolute iron deficiency	Systemic Iron Deficiency (SID)	Low ferritin (<100 ng/mL) and/or low TSAT (<20%), reflecting insufficient systemic iron.	[5]
Functional iron deficiency	Iron-Restricted Erythropoiesis (IRE)	Standard or high ferritin, but TSAT <20%, usually due to hepcidin-mediated restriction.	[5,11]
Hemochromatosis / Siderosis	Iron Overload (Primary / Secondary)	Pathological accumulation of iron in tissues; primary (genetic) or secondary (transfusion-related).	[6]
Labile plasma iron	Non-Transferrin Bound Iron (NTBI)	Circulating iron not bound to transferrin, often elevated in overload states.	[5]
Serum Fe	Serum Iron	Concentration of iron bound to transferrin in circulation.	[12]
Transferrin Saturation Index (TSI)	Transferrin Saturation (TSAT)	Ratio of serum iron to TIBC, expressed as a percentage.	[13]
Ferrous (Fe2+)	Iron(II)	Preferred IUPAC name for ferrous ion (Fe²⁺).	[7]
Ferric (Fe3+)	Iron(III)	Preferred IUPAC name for ferric ion (Fe³⁺).	[7]
Anaemia of chronic disease	Anaemia of Inflammation	Anaemia mediated by inflammatory cytokines and hepcidin, leading to restricted iron availability despite adequate stores.	[10]

Beyond nephrology and hematology, the implications of standardized nomenclature extend to multiple specialties, including oncology, cardiology, infectious diseases, and maternal-fetal medicine. Each field employs unique thresholds and interpretations of iron indices, often derived from context-specific pathophysiology. Adopting a unified framework ensures that data from diverse disciplines can be meaningfully compared, particularly in multi-center and multidisciplinary research. This convergence facilitates meta-analyses, guideline development, and cross-specialty education, thereby fostering a more integrated understanding of iron biology in health and disease.

Harmonizing nomenclature supports precision in clinical research, enhances the comparability of laboratory indices across studies, and prevents miscommunication between clinicians, laboratory scientists, and policymakers [14]. Explicitly noting both former and new terminology would help researchers and clinicians better interpret historical literature while applying the most accurate and contemporary definitions in current practice. Standardized terminology also strengthens education and training, ensuring that students, trainees, and healthcare staff learn consistent, precise terms, thereby promoting effective communication and knowledge translation across clinical and laboratory settings.

From an educational standpoint, harmonized terminology offers a foundation for consistency across curricula in medicine, nursing, and biomedical sciences [15]. Integrating the updated lexicon into undergraduate and postgraduate programs will ensure that emerging professionals communicate with precision and align with international standards. Additionally, translating this framework into multilingual educational materials can enhance global reach, particularly in regions where local terminology or legacy translations have perpetuated outdated terms.

Despite a consensus on updated terminology, implementing standardized nomenclature across healthcare settings remains a challenge. Laboratories need to revise reporting templates, clinicians must adapt to new terminology, and educators must update curricular for trainees. Resistance may arise from familiarity with older terms or perceived complexity in translating chemical and clinical nomenclature into daily practice [9]. 

Implementing harmonized terminology requires a structured, multi-stakeholder approach. Professional societies and guideline committees should formally endorse the standardized lexicon and incorporate it into diagnostic algorithms, clinical decision-support tools, and continuing education programs. Laboratories can play a pivotal role by aligning their reporting formats and reference ranges with updated definitions, accompanied by interpretive comments that bridge chemical and clinical perspectives. Collaboration between electronic health record (EHR) vendors, laboratory information system (LIS) developers, and regulatory bodies can further facilitate automated mapping of old-to-new terms, reducing the risk of misclassification and ensuring consistency in patient records.

Successful implementation will require targeted educational strategies, integration of standardized terms into electronic health records and laboratory information systems, and endorsement by professional societies. Tailored approaches that consider local context, resources, and training needs will be essential to ensure widespread adoption and to maximize the benefits of harmonized terminology for research, clinical care, and education.

Looking forward, sustained global collaboration will be essential to maintain and evolve this nomenclature as scientific understanding deepens. International organizations such as the World Health Organization (WHO), the International Federation of Clinical Chemistry (IFCC), and the International Committee for Standardization in Hematology (ICSH) could establish joint task forces to oversee updates and ensure equitable adoption across high-, middle-, and low-income countries. Digital health platforms and artificial intelligence tools could further support this harmonization by automatically standardizing terminology in laboratory reports, manuscripts, and clinical registries [16].

To sum up, iron is a single element, yet the language used to describe it in medicine has long been fragmented and confusing. Inconsistent terminology has hindered clear communication, accurate diagnosis, and the synthesis of research evidence. The updated, harmonized nomenclature provides a unified framework that bridges chemical precision, laboratory reporting, and clinical interpretation. Adopting this framework is more than a semantic exercise; it is a practical necessity for clinicians, researchers, educators, and policymakers alike. Embracing standardized terminology enables the medical community to improve patient care, strengthen research comparability, and ensure that future generations of healthcare professionals learn and communicate with clarity. The challenge now lies not in defining the terms, but in integrating them into practice, where the benefits of harmonized terminology can be fully realized.
